# Impact of social risk factors on TF-CBT engagement and strategies to mitigate the impact: A qualitative analysis

**DOI:** 10.1371/journal.pmen.0000499

**Published:** 2026-04-01

**Authors:** Clara Johnson, Sharon Kiche, Anne Mbwayo, Daisy Anyango Okoth, Omariba Anne Nyaboke, Anna Testorf, Priya Dahiya, Shannon Dorsey

**Affiliations:** 1 Department of Psychology, University of Washington, Seattle, Washington, United States of America; 2 Department of Psychiatry, University of Nairobi, Nairobi, Kenya; 3 TF-CBT Trainer/Mental Health Consultant, Bungoma, Kenya; PLOS: Public Library of Science, UNITED KINGDOM OF GREAT BRITAIN AND NORTHERN IRELAND

## Abstract

Social risk factors, or adverse conditions in which people are born, grow, live, and age (1), contribute to greater mental health problems (2,3) and lower treatment engagement worldwide (4). The prevalence and impact of social risk factors (SRFs) are particularly pronounced in low-to-middle income countries (LMICs); however, strategies to address SRFs alongside mental health treatments have not been systematically studied. Building on an NIMH-funded project (BASIC; 5), we invited participants ranging from children and guardians who received and lay counselors that delivered an adapted form of Trauma-Focused Cognitive Behavioral Therapy (TF-CBT; 6) in western Kenya to complete qualitative interviews. In these interviews, we examined both the experience of SRFs and how lay counselors address SRFs within TF-CBT. Results from the qualitative analysis reveal that in the face of SRFs, counselors were already addressing SRFs, though typically by using their own personal resources. Participants also identified a variety of strategies that do not require extensive financial support. Overall, the experience of SRFs and strategies to address SRFs reported in this paper may support the engagement in and effectiveness of evidence-based practices in Kenya and globally.

## Introduction

### Inequity of mental health access worldwide

Over 13% of the world’s population lives with a mental disorder [1,2]. Although most of the world’s population reside in low-to-middle-income countries (LMICs; [3]), access to mental health care in LMICs is low (5–21%; [4]). Low access to mental health care can be attributed to limited availability of providers and less funding for mental health services in LMICs [5]. When treatments are available, they are often unaffordable and not appropriate for the context, limiting the feasibility, acceptability, and effectiveness of the care [6]. Of those who seek treatment, few (3.7-11.4%) receive minimally adequate care. The disparities among youth seeking mental health care within LMICs are even wider due to under-representation in treatment development and evaluation studies, lack of youth mental healthcare providers, and higher prevalence of mental health disorders [7,8]. In addition, youth in LMICs experience high rates of social risk factors (SRFs). SRFs are defined as the adverse social determinants of health, or conditions in which people are born, grow, and live [9]. These SRFs, compounded by recent historical impacts (e.g., the COVID-19 pandemic), can have negative impact on mental health and impede access to mental health care [10–12,13]. Despite the prevalence of mental health difficulties, SRFs, and concentration of youth within LMICs, the majority of global mental health research and implementation is conducted in high income countries [8]. It is important to acknowledge that the term, LMICs, covers a wide variety of contexts in which needs and resources differ greatly; however, we use this term to capture contexts in which access to mental health providers is low and prevalence of SRFs and mental health burden is high.

### Impact of parental death on mental health prevalence

A sub-population of youth concentrated within LMICs who experience high rates of mental health difficulties and SRFs are children orphaned by one or both parents. As of 2014, 47.5 million children were estimated to be orphaned by at least one parent in sub-Saharan Africa, making up about a third of the reported orphaned children worldwide [14,15]. At the same time, roughly 1.8 million of these children lived in Kenya – 15% of whom lost two parents [16]. As of 2023, it is estimated that 700,000 children in Kenya lost a parent to HIV, contributing to about 39% of total parental deaths [17]. Children orphaned by one or both parents are at a high risk of experiencing depression, prolonged grief, anxiety, post-traumatic stress, and behavioral problems [18–20]. In addition, the experience of parental death is associated with several risk factors for mental health difficulties, including poverty, housing insecurity, difficulty attending school, lack of social support, and increased vulnerability for abuse and stigma – particularly related to HIV (e.g., [21–24]. Furthermore, in a study of counselors who delivered trauma-focused evidence-based practices (EBPs) to children orphaned in Kenya, 81% of guardians who participated in the therapy reported that their child went to bed hungry some or all of the time in the past fourteen days [25].

### Social risk factors

Social risk factors (SRFs) can include, but are not limited to, food insecurity, water insecurity, insufficient economic needs (e.g., school fees for children), and experiences of discrimination [9]. More broadly, SRFs can be considered a sub-category of social determinants of health/mental health, which include both the protective and risk factors that impact an individual and their health [26]. We use the term, ‘social risk factors’ to identify the social determinants of health/mental health that have a negative impact on individuals and their response to mental health treatment. The Center for Disease Control and Prevention (CDC) considers SRFs within five main domains: “economic stability, education access and quality, healthcare access and quality, neighborhood and built environment, and social and community context” [27]. Research indicates that SRFs are associated with a wide range of mental health problems in high-income countries [28,29] and LMICs [30,31]. Counselors in the United States recognize the importance of addressing SRFs, yet do not feel as though they received adequate training to address these factors in their provision of mental health care [32–34]. Moreover, many researchers have reported calls for training counselors to address SRFs in LMICs [35–38]. Others have called for integration of SRFs within mental health assessment and evaluation [38–40]. Initial research outlining guidelines for addressing SRFs do exist [39,41,42], yet most of these frameworks are situated within high income contexts.

### Addressing social risk factors in LMICs

Mitigating the effects of SRFs is especially important among children who are orphaned in LMICs. Although SRFs often co-occur and contribute to mental health problems in LMICs [43,44], existing strategies to improve access and engagement (i.e., session attendance, homework adherence, strong relationship between client and counselor, and overall participation in therapy; [45]) in EBPs have not typically addressed or mitigated SRFs. In a study that examined guardian and lay counselor perspectives on the implementation of a trauma-focused EBP for youth in Kenya, 36% of the guardians engaged in the EBP explicitly recommended that the EBP offer support for SRFs [46]. In addition, lay counselors in LMICs [47] reported feeling ill-equipped to discuss and address SRFs, highlighting the need to develop strategies that can be integrated with EBPs to target this issue [48–51]. Within LMICs, there are broader calls to address SRFs through programs like cash transfers. For example, [52] investigated the existing cash transfer programs that provide economic empowerment through financial literacy and funding. However, Lund (2023) explains that these programs typically require significant policy and governmental support and may not be feasible in some contexts. An ongoing study, “ALIVE”, is one example in which a combination of emotion regulation and economic intervention is grouped together as a program for adolescents in Bogota, Cape Town, and Kathmandu [53]. ALIVE is the first of its kind to combine mental health care with strategies to address SRFs, though still relying on cash transfers and significant funding. Further research is needed to identify ways to mitigate the impact of SRFs within mental health care that do not require substantial financial support.

### Adaptations to EBPs

In order to address SRFs within EBPs, we draw on the literature on how to effectively adapt EBPs while maintaining fidelity-- whether and how well the EBP is delivered according to the protocol [54]. Modifying EBPs is essential when planning implementation of an EBP in a context that misaligns with the initial research context and has more comorbidities, complexities, or limited resources [55–59]. As most youth mental health research is concentrated in high income countries, adaptation is particularly key in LMICs. Development of a new intervention is another solution, though requires substantial money and time [60]. In a recent overview of adaptations of EBPs, Stirman (2022) [61]describes the importance of designing fidelity-consistent adaptations, ones that keep the core components of an EBP. Marques et al. (2019) [62] found that among community-based providers of a trauma-focused EBP, those who engaged in fidelity-consistent adaptations towards the beginning of treatment saw larger decreases in posttraumatic stress and depression than those with fidelity-inconsistent adaptations. To guide the fidelity-consistent adaptations, Stirman (2022) references existing frameworks that can guide decision-making regarding adaptations, like the FRAME (Framework for Modification and Adaptations; [[59,63]]). In addition to frameworks, Stirman (2022) [61] suggests continuous external support to ensure consistent use of fidelity-consistent adaptations (e.g., through supervision). Integration of existing frameworks and supervision can support the development and integration of strategies to mitigate the effect of SRFs alongside an EBP.

### Overview of current study

The current study builds on an NIMH-funded study of the implementation practices and policies that support TF-CBT in schools delivered by lay-counselors (teachers and community health volunteers) within the context of Bungoma County, Kenya [64]. We first recruited children, guardians, and counselors who received or delivered TF-CBT for qualitative interviews to guide the strategy development. Qualitative interviews evaluated the types of SRFs witnessed by participants, how the SRFs impacted engagement in TF-CBT, and how counselors were already addressing SRFs in therapy. All participants were asked how the TF-CBT training could better equip counselors to address SRFs, taking the financial and logistical burden away from counselors. Through qualitative analysis that privileged the perspectives of the clients and providers of TF-CBT in western Kenya, we aim to inform development of strategies to address SRFs within youth mental health care.

In order to address SRFs within EBPs, we draw on the literature on how to effectively adapt EBPs while maintaining fidelity-- whether and how well the EBP is delivered according to the protocol [54]. Modifying EBPs is essential when planning implementation of an EBP in a context that misaligns with the initial research context and has more comorbidities, complexities, or limited resources [55–59]. To guide the fidelity-consistent adaptations, Stirman (2022) references existing frameworks that can guide decision-making regarding adaptations, like the FRAME (Framework for Modification and Adaptations; [58,62]). In addition to frameworks, Stirman (2022) suggests continuous external support to ensure consistent use of fidelity-consistent adaptations (e.g., through supervision). As a part of the current study, we utilize existing frameworks and supervision to support the development and integration of strategies to mitigate the effect of SRFs alongside an EBP.

## Method

### Ethics statement

Ethics approval was provided by Kenya Medical Research Institute, the University of Washington and Duke University institutional reviewer boards. Children participate in interviews as a part of existing research procedures of the parent study, so the authors followed procedures already set in place by the parent study institutional review board (Kenyan Medical Research Institute). Children provided assent, and their guardians provided informed verbal consent prior to participation. The guardian and counselor interviews were approved by the University of Washington institutional review board. Each guardian and counselor participant provided informed written consent to participate in the interviews.

### Parent study

The project builds off a Hybrid Type II Implementation-Effectiveness trial, “Building and Sustaining Interventions for Children (BASIC): Task Shifting Mental Health Care in Low Resource Settings”. In the BASIC trial, teachers and community health volunteers implemented a culturally adapted version of TF-CBT [65] in schools in western Kenya called *Pamoja Tunaweza (PT;* [64,25,65,66]). Through a well-established partnership between a Kenyan non-governmental organization, Ace Africa, and two large universities in the United States, PT was initially designed by and for the community and found to be effective at decreasing symptoms of post-traumatic stress and prolonged grief [25]. To build on the effectiveness of PT, the BASIC study aimed to identify implementation practices and policies that contributed to the implementation and sustainment of PT within the health and education sectors. In BASIC, PT was delivered by lay counselors (community health volunteers and teachers) to children aged 11–14 who lost one or two parents. Community health volunteers (CHVs), as they typically deliver health services within the community, represented the ‘health sector’, and teachers represented the ‘education sector’. Although the sectors were considered separate within the BASIC trial, all PT groups regardless of sector delivered PT within schools due to community partner requests. BASIC followed a partial stepped wedge cluster randomized design in which the teachers in 40 schools and CHVs within the 40 surrounding communities were randomly assigned to seven sequences of training and implementation of PT. All research activities (e.g., counselor PT trainings, children and guardians engaging in PT) in the parent study occurred between 2018 and 2024 and were approved under the Duke University Institutional Review Board and the Kenya Medical Research Institute.

Pamoja Tunaweza (PT) was delivered to children and their primary guardian who experienced parental loss and heightened levels of posttraumatic stress and/or prolonged grief [25]. PT in the BASIC trial follows an eight-session group-based format in which two counselors deliver material to children, and one counselor delivers material to guardians. The topics of the groups follow the core components of TF-CBT including psychoeducation, relaxation, affect modulation, cognitive coping, the trauma narrative, in vivo exposure, conjoint guardian and child groups, enhanced safety, and four grief components. Although most components were delivered in group format, the trauma narrative (i.e., imaginal exposure) components of PT were delivered individually [25]. Adaptations to TF-CBT include separation of girls and boys, use of group format, and tailored language and examples that are relevant to the Kenyan context. TF-CBT supervisors employed by Ace Africa, were trained as trainers and supervisors through a Train-the-trainer model, provided training and follow-up supervision to all CHVs and teachers in the BASIC trial.

### Participants

Participants included 12 PT counselors who were currently providing PT (six CHV counselors; 6 teachers), 20 guardians, and 20 children (10 boys; 10 girls) who received PT through BASIC during or after 2020 for recency. To ensure a variety of voices were represented in the interviews, we invited three CHVs and three teachers from urban schools and three CHVs and three teachers from rural schools to participate in the interviews. To include participants who engaged in PT at varying levels, we invited 10 guardians and 10 children that attended less than or equal to four of the eight PT sessions. Of note, although some guardians and children attended lass than or equal to four sessions, counselors provided these participants with review of missed material outside of session time. PT supervisors have noted that CHV and teacher counselors encounter similar SRFs among children enrolled in PT, thus allowing for inclusion of both counselor types. Based on qualitative research saturation standards, we included at least 12 interviewees per participant type to accurately capture the voice of both teachers and CHVs [67,68]. Recruitment began on February 15, 2023 and all recruitment and interviews were completed on April 20, 2023.

### Procedures

The qualitative interview guide was developed through three web-based meetings with PT supervisors, a Kenyan psychologist, and trained interviewers from Ace Africa (S1 Text). The guide development began with orientation to specific requests of PT children, guardians, and counselors from past qualitative studies [69,70]. Developed interview questions used the wording from past counselors and PT recipients (e.g., “non-mental health needs/hardships” rather than SRFs; [69]. For child and guardian interviews, the team built on research that already identified common SRFs among this population [51,70] and designed initial questions to examine children and guardian perspectives on experiences specifically within the PT context. For child interviews, the team developed two questions that identify if and how PT counselors address SRFs and how PT could better address SRFs during sessions. For counselor interviews, the team developed questions that assessed barriers and facilitators counselors faced in addressing SRFs in PT.

Trained male and female interviewers that had at least a bachelor’s degree interviewed ten guardians and ten children that attended fewer than or equal to four of the eight PT sessions to capture the voice of children and guardians who may have had more barriers to attend PT than others. Contact began with written informed consent and description of incentives provided for participation. Among child participants, interviewers obtained guardian consent and child assent. Each interview lasted 15 minutes (children), 30–45 minutes (guardians), and 45–60 minutes (counselors). Out of the 80 total children who completed PT between 2022–2023 as a part of the BASIC trial, 20 children were randomly selected to respond to two additional questions alongside the existing interviews all 80 children completed as a part of the BASIC trial. The research team only included two additional questions to limit burden on the children as they also completed additional qualitative questions at the same timepoint for BASIC. Interviews were conducted in Kiswahili, minimizing the influence of the colonizer’s language (i.e., English; [71]) unless the participant preferred English. Following BASIC study incentives, the amount given to participants was commensurate with interview length (guardian: 250 ksh/$2.50 USD; counselor: 500 ksh/$5 USD). All guardian and counselor interviews were approved by the University of Washington institutional review board. Interviews occurred at participant homes and schools depending on participant preference. Interviewers read scripted questions to participants and provided prompts when necessary.

During each interview, an interviewer served as a scribe and included detailed verbatim notes of each interview response. The notes were uploaded to a secure data platform for review by the research team. To push against inequities inherent in global research, the qualitative guide development was based on the Kenyan research team’s lived experience and expertise and the first author’s experience with qualitative methods in crafting questions that assess content areas brought forward. Interview guides were created in Kiswahili and English (as some teacher counselors expressed preference for English). We utilized translation/back translation by different individuals to ensure content consistency across languages. Once interviews were drafted, the first author and research team reviewed them for adherence to semi-structured interview standards (e.g., questions are not leading). Interviewers participated in a refresher on qualitative interviewing and were invited to bring forth their expertise and skills, given that interviewers had been trained in qualitative methods and conducted qualitative interviews in previous BASIC projects.

### Analysis

Interviews were recorded and detailed notes were taken during the interview and before analysis. To inform the workshop and training, the all-female coding team (PT supervisors, the first author, and a Kenyan research team member with Kiswahili fluency and lived experience in Kenya) first used *rapid qualitative analysis* to code the semi-structured interviews [72,73]. Rapid qualitative analysis allows for a shorter period of analysis over a shorter period of time compared to other forms of qualitative analysis (e.g., thematic analysis), given the quick turnaround between the interviews and the workshop due to the BASIC study timeline and the school schedule in the research context [74,75]. However, following the workshop and training, a decision to conduct a more *detailed deductive thematic analysis* was made based on discussions with community partners and a desire for more analysis that captures the nuance not identified during rapid qualitative analysis. We followed thematic analysis guidance from Bruan and Clarke (2006).

The coding team consisted of five members. Most coders (3/5; 60%; OAN, DAO, SK) were born in Kenya and were multilingual, fluent in Kiswahili and English, and two coders (AT, CJ) were born in the United States and fluent in only English. Two coders held a master’s degree, two coders held bachelor’s degrees, and one coder was an undergraduate research assistant. Prior to the onset of thematic analysis, two independent coders listened to an English and Kiswahili interview to determine whether the level of detail within the uploaded interview notes accurately represented the information included in the recorded interview audio files. The notes were found to reliably capture the recorded interview audio. Thus, following research that indicates data collected via scribe is a feasible and reliable alternative to transcription when resources are limited [76] and can eliminate potential bias introduced by transcripts [77], we coded directly from the detailed notes rather than transcriptions. Notes that were completed in Kiswahili were not translated as three raters were multilingual, and therefore, focused on analyzing notes in Kiswahili. No transcripts were written as a part of this study. The first author (CJ) and co-author (SK) trained the team of coders in thematic analysis through in person and virtual meetings. CJ and AT next reviewed all interviews completed in English independently, developing themes and codes that arose from the data (*n* = 4) within an initial codebook. SK piloted the codebook and reviewed the same four English interviews along with two Kiswahili interviews completed with counselors and two Kiswahili interviews completed with guardians, adapting it as needed. All coding was completed using Microsoft Excel.

After the initial codebook was developed and piloted, all interviews were coded by at least two separate coders and any discrepancies were discussed and resolved, coding to consensus. The codebook was adapted, when necessary, throughout coding, and when additional themes emerged, members of the coding team reviewed all interviews to ensure each new theme was captured. All coders collaboratively developed themes by which the codes could be grouped. The methods and results reported in this paper align with the COREQ guidelines (Consolidated Criteria for Reporting Qualitative Research; See S1 COREQ Checklist; [78]). In a subsequent study, the results were member checked with 25 other lay counselors enrolled in the BASIC study.

## Results

### Overview

Overall, 12 lay counselors (6 CHV, 6 teacher), 20 guardians, and 20 children were invited and interviewed in early 2023. Ten boys and ten girls participated in the interviews and were on average 14 years old (SD = 1.14 years) at the time of interviews. Most guardians were female (85% female, 15% male) and on average 40 years old (SD = 10.25) at the time of interviews. Sixty-five percent of guardians were biological parents of the children (*n* = 13), with the remainder grandparents (*n* = 4) or maternal aunts (*n* = 2). Eighty-three percent of the CHV counselors were female (*n* = 5) and all received a secondary education as their highest level of education. CHVs were on average 46 years old (*SD* = 9.73) at the time of interviews. Sixty-seven percent of the teacher counselors were female (*n* = 4), and their highest level of education ranged from a certificate (*n* = 3), master’s degree (*n* = 2), and secondary education (*n* = 1). Teachers were on average 44 years old (*SD* = 6.72) at the time of interviews. Prior to enrollment in the BASIC study, 33% of CHVs and teachers received training in mental health care and 50% of CHVs and teachers provided mental health care. No participants declined or dropped out of the study (Table 1).

**Table 1:** Participant demographics.

Demographic Variable	CHV Counselors (*n* = 6)	Teacher Counselors (*n* = 6)	Guardians (*n* = 20)	Children (*n* = 20)
Age [*M* (*SD*)]	46 (9.7)	44 (6.7)	40 (10.3)	14 (1.1)
Female (%)	83.3%	67.7%	85.0%	50.0%
Male (%)	16.7%	33.3%	15.0%	50.0%
Completed treatment*	N/A	N/A	100.0%	100.0%

*Note.* *For those who attended 4 or fewer sessions, counselors provided individual review sessions covering the missed content. Thus, all children and guardians completed treatment. CHVs = community health volunteers, *M* = mean, *SD* = standard deviation, N/A = not applicable.

### Social risk factors

Guardians and counselors described many social risk factors (SRFs) that they witnessed within the context of Pamoja Tunaweza (PT). All SRFs reported are in Table 2. All counselors reported lack of uniforms, lack of school fees (e.g., money needed for exams and/or enrollment), and lack of food. All guardians similarly reported lack of school fees, and most reported lack of food.

**Table 2:** Social risk factor types.

Theme	Social Risk Factor
Daily Living	Lack of food
	Lack of home clothes
	Lack of housing necessities
	Lack of shoes
	Lack of sufficient accommodation
Financial	Lack of money and resources
Mental Health/Attitudes	Lack of guardian support
Physical Health	Lack of medical resources
	Lack of sufficient hygiene products
School	Lack school fees
	Lack of school supplies
	Lack of uniforms
Stigma	Discrimination

### Impact of social risk factors

Guardians and counselors were asked how SRFs impacted children, guardians and specifically their service utilization of PT. Counselors and guardians reported that SRFs negatively impacted both child and guardian PT participation, engagement, and attendance. In addition, poor child and guardian self-esteem and mental health were observed by guardians and counselors alongside the experience of SRFs. For example, a counselor reported:

When you’re teaching and you find one or two children are in torn uniforms, when they come, they tend to sit by themselves. They won’t sit with others freely. They’re somehow withdrawn and they feel embarrassed. It makes them feel less.

A counselor added:

Money is everything. A child who comes from very far, sometimes walking distances coming to school and back. They reach when very tired… Sometimes they don’t have money to buy lunch. When it comes to sessions, they are so exhausted, weak, they can’t contribute comfortably like the others.

A guardian also noted:

It’s up to the parent to look [for means to feed] the family, now, when it reaches the time when PT teachers need them in school, the parent misses the session because they’ve gone to look [for means to feed the family]. (Kiswahili: Mzazi inabidi aende atafutie familia sasa ikifika wakati walimu wa PT wanawahitaji shuleni itabidi akose kuhudhuria kwa sababu ameenda kutafuta.)

### Barriers in addressing SRFs

Both guardians and counselors were asked what barriers impeded the counselors’ abilities to address SRFs. Guardians and counselors agreed that guardians expected PT to provide financial support, and this expectation made it difficult for counselors to help children mitigate the effect of SRFs without providing money or physical resources. Most counselors also reported that a lack of money and resources among counselors presented as a barrier to addressing SRFs. A counselor stated, “The barrier is that even the counsellors don’t have enough money…We don’t have enough funds to provide for them and for all their needs”. Another counselor reported that the main barrier was, “Lack of money. Us counselors, we wish we had money to support these children fully for supports like pair of shoes or shirt yearly [uniforms]”. Further, we emphasize that the burden should not be placed on counselors to provide money or resources, and thus, the PT program dissuaded counselors from using their own funds to help children.

### Communication about SRFs

Counselors were asked about how guardians and children shared information regarding SRFs with them. Over two thirds of counselors reported that the structure and components of PT, observation of how children lived, and children and guardians’ willingness to discuss SRFs helped counselors understand the SRFs experienced by children. One counselor stated, “When it came to the narrating kumbu kumbu ugumu [trauma narrative], that’s the time they were able to narrate to us the hardship they’re going through”. Another counselor reported, “The parents were so free with me. They would tell us the challenges they were facing and how they were dealing with them. The parents themselves were able to discuss how best to address those challenges.” (Kiswahili: Wazazi walikuwa so free kwangu. Walikuwa anatueleza ugumu wanazopitia na jinzi wanavyoeza kuendelea kukumbana nazo. Hata wenyewe wakajadiliani ni namna gani wanaezafanya).

### How counselors were *Already* addressing SRFs in PT

Children, guardians, and counselors were asked what counselors were already doing to help mitigate the effect of SRFs within PT. Many strategies were reported as already being used (see Table 3, the highlighted column “used” to for those reported as used already). However, children, guardians, and counselors reported noticing the following strategies already being used, often via personal counselor expenses: providing school uniforms, providing school supplies, providing food, providing financial support, providing clothing, and collaborating with administration and teachers to better support children enrolled in PT. For example, a counselor stated, “Sometimes I could go for my own pocket”. A guardian expanded on the type of support counselors would provide: “They catered for the uniforms, books. They helped out a little with the fees.” (Kiswahili: Walishughulikia uniform, vitabu. Walisaidia fee kidogo). Another counselor described the collaboration with administration:

**Table 3:** Strategies to address SRFs.

Strategy	Actor	Resources	Used	Suggested
Coordinating and Collaborating with External Resources				
Collaborate with administration to support children enrolled in PT	School	Moderate		
Pool community resources	Community	High		
Provide guardians with jobs	Community	High		
Provide referrals	Counselor	Low		
Mental Health/Attitudes				
Encourage guardians	Counselor	Low		
Provide continued mental health services	Counselor	High		
Intervention Characteristics				
Extend PT (scope and session frequency)	PT	Moderate		
Expect to adapt PT to the needs of enrolled children	PT	Low		
Increase awareness about PT	PT	Low		
Increase counselor training	School	Moderate		
Increase guardian trust in counselor and PT	Family	Low		
Provide peer counseling	Family	Moderate		
Provide vignettes of children who have addressed SRFs within PT successfully	PT	Low		
Daily Living				
Improve accommodation	Community	High		
Provide food	Multiple	Moderate		
Provide housing necessities	Multiple	Moderate		
Provide sufficient clothing	Multiple	Moderate		
Teach life skills	Counselor	Low		
Financial				
Provide financial support	Multiple	High		
Pay school fees	Multiple	High		
Teach economic empowerment	Counselor	Low		
Physical Health				
Provide access to physical healthcare	Counselor	Moderate		
Provide health education	Counselor	Low		
Provide hygiene products	Counselor	Moderate		
School				
Allow children to do homework in school	School	Low		
Provide educational school supplies	Multiple	Moderate		
Provide school uniforms	Multiple	Moderate		
Stigma				
Encourage guardians	Counselor	Low		
Teach acceptance of children acceptance	Multiple	Low		

*Note.* Shaded boxes indicate that these strategies were highlighted in at least two qualitative interviews. PT = Pamoja Tunaweza; SRF = Social Risk Factor. Resource levels were operationalized as follows: Low = strategy can be implemented with existing resources and within school day; Medium = strategy requires some additional funding, donation, and time outside a typical workday; High = strategy requires a substantial amount of funding, donation, and time outside a typical workday.

Sometimes when the headteacher and Deputy send children home for school levies we talk to the administrations to at least pardon them and understand them, meanwhile they will bring the money later or pardon them they just get into the system without pay.

### Suggested strategies to address social risk factors within PT

To reduce the burden experienced by counselors, especially considering that all counselors reported witnessing another counselor giving PT children financial support, all participants were asked how PT can take on the burden of addressing SRFs. Children, guardians, and counselors agreed on the following tangible strategies that require funding: providing sufficient clothing, school uniforms, hygiene products, food, school supplies, access to physical healthcare, school fees, and children’s accommodations. Despite many of those strategies requiring substantial financial support, many participants suggested strategies that do not necessarily require money: teaching acceptance of children experiencing parental loss and encouraging guardians to support their children. In addition, guardians and counselors suggested teaching life skills, economic empowerment, health education within PT, increasing the scope of PT training includes, and providing continued mental health counseling beyond PT, among other strategies (See highlighted column “suggested” in Table 3 for suggested strategies). Many of the strategies that participants suggested could be utilized by PT were already being utilized by counselors. A counselor provided additional detail regarding economic empowerment: “Economic empowerment to the locals. Activities that guardians can engage in to be economically empowered. For example, joining groups and doing things like planting a [tree] nursery”.

### Impact of addressing SRFs

Lastly, guardians and counselors considered the impact they witnessed when counselors used the strategies to address SRFs within PT. Counselors and guardians overlapped in many of the impacts they noted. Specifically, most counselors and all guardians reported improved child mental health. Most guardians and counselors also reported improved child and guardian self-esteem and guardian mental health after addressing SRFs. Counselors and guardians noticed that after addressing SRFs, the SRFs were generally mitigated, guardians increased their support for PT children, children attended school more consistently, and children improved academically. Most counselors noted that children were more engaged and attended more PT sessions. Overall, addressing SRFs seemed to positively impact children and guardian mental health and improve children’s engagement in school and in PT. A guardian reported:

When we were in class, when we saw what the counselor did for this child, it encouraged us. So, it extended to children and parents. PT has power because we saw it working. The children saw that when they have a problem, they would share and get help. (*Kiswahili: Ndio. wakati tulipokuwa kwa darasa, tulipoona kile mshauri alifanya kwa huyu mtoto, kilitutia moyo. Kwa hivyo iliextend kwa watoto na wazazi. PT has power because tuliona ikifanya kazi. Watoto waliona kumbe wakiwa na shida wanaeza share na kusaidika).*

In addition, a counselor stated, “[Addressing social risk factors] contributed also to their mental health healing. You find that now a child has raised up her/his self-esteem”.

## Discussion

To address barriers in accessing and engaging in mental health care, the current study explored the impact of SRFs on mental health and treatment engagement and strategies that can be integrated into group interventions for children to address the SRFs. There was a wide range of SRFs reported by guardians and counselors, and participants noted negative impact of SRFs on mental health, self-esteem, and engagement in therapy. Given the negative impact that counselors saw, it is important to note that many counselors were already engaged in strategies to address SRFs alongside PT. However, most strategies relied heavily on a counselor’s personal financial resources. Lay counselors in the study context were already providing mental health support outside their typical work duties and should not hold the financial burden of addressing SRFs. Guardians and counselors similarly identified the main barrier in addressing SRFs in therapy as the lack of money and resources among counselors. Providing personal resources to address SRFs presents significant ethical concerns and increases burnout among therapists [79,80]. Moreover, if counselors do not have options for addressing SRFs and thus use their personal funds, these situations contribute to inequitable practices adding undue burden on counselors in lower resourced settings and unequal support of youth dependent on counselor resources. To support ethical practices, there is a to identify strategies that are feasible, do not require money, and rely on the existing resources and strengths within the community.

The adverse effect of SRFs on children and guardian well-being and therapy engagement underscores the importance of selecting strategies that will likely have a wide and sustained impact. Overall, participants noted positive impact on child and guardian mental health and PT engagement when counselors’ delivered strategies to mitigate the effects of SRFs within PT. As a member on the research team stated in a team meeting, ‘while it may seem easier to address the immediate need (i.e., giving a someone who is hungry a fish), it is more important to teach this person how to address their own need in the future (i.e., teaching someone how to fish for their own food)’. In considering the potential sustained impact of strategies, we should consider whether the strategy targets a common SRF reported in the community and relies on existing resources and supports in the community. The impact of the strategy is likely dependent on the context and what is needed, so we are hesitant to identify strategies that have the biggest impact across multiple sites with different needs. Instead, we encourage considering which SRFs are reported in a specific community and the potential impact anticipated on SRFs before utilizing any particular strategy.

### The FRAME for selecting strategies

Overall, the results indicate that the needs, resources, and strategies within each site differ, and a context-dependent approach to selecting strategies is warranted. The evaluation of impact and feasibility will also depend on these context-specific considerations. We look to existing frameworks to support these context-specific decisions. Stirman et al. (2013) first developed and then expanded the ‘FRAME’ [59], a framework for reporting adaptations to evidence-based practices, to support researchers and practitioners in reporting and assessing adaptations made to EBPs. The FRAME considers what the adaptation is and when, why, how, by whom, and *for* whom is the adaptation made. Although the FRAME was initially designed to assist in the reporting of adaptations, we propose using the FRAME to guide which strategy should be used in which context and assess the potential impact and feasibility of strategy implementation. Given the difference in which we use the FRAME, we adjust the language and ordering of the questions to fit the context of our work in the adapted-FRAME (see Table 4).

**Table 4:** Adapted questions from the framework for modification and adaptations.

Original FRAME Questions	Adapted-FRAME Questions
(1)When did the adaptation occur?	(6)When will the adaptation be made in the implementation process?
(2)Was the adaptation planned/proactive or unplanned/re-active?	(3)Has this adaptation been made proactively or re-actively before? What are lessons learned?
(3)Who participated in the decision to modify?	(8)Who is involved in executing the adaptation? Will all perspectives be considered?
(4)What is modified?	(4)Is the adaptation changing the content, how the treatment is delivered, the training in the treatment, or the treatment implementation?
(5)For whom/what is the adaptation made?	(7)Who will be impacted by the adaptation?
(6) What is the nature of the content adaptation?	(2)What type of adaptation is this?
(7)What is the relationship to fidelity/core elements?	(5)Will the adaptation change the core elements of PT?
(8)What were the reasons for the adaptation?	(1)Why do you need to make an adaptation?

*Note.* Questions are adapted from the Framework for Modification and Adaptations (FRAME; [59,63]). PT = Pamoja Tunaweza.

### Applying the FRAME to developed strategies

#### Leadership collaboration.

Based on context knowledge and past knowledge, we presented two strategies through the adapted-FRAME questions. First, many counselors and guardians reported that working with the school administration to find strategies to address lack of school fees, torn uniforms, and lack of food is an important strategy. (1) Why do you need to make an adaptation? This adaptation is in response to one of the most reported SRFs, school fees, which can have impact on other financial barriers to engagement in PT, school attendance, and overall child wellbeing. (2) What type of adaptation is this? This adaptation is a contextual adaptation in which the individuals involved in the implementation of PT are adjusted (i.e., counselors work with leaders and administrators to support children). (3) Has this adaptation been made proactively or re-actively before? What are lessons learned? Counselors reported previously engaging with administration to waive or postpone school fees in response to the SRF, which has allowed children to attend PT even if they did not pay all school fees. Counselors also reported seeing improvements in mental health and engagement following collaboration with administration. More broadly, implementation leadership, or the support leaders provide in the implementation of an EBP, is tied to successful EBP implementation in the literature, which further supports this strategy [81,82]. (4) Is the adaptation changing the content, how the treatment is delivered, the training in the treatment, or the treatment implementation? The adaptation is changing how the treatment implementation is supporting children as school leadership will help remove barriers to attending PT groups. (5) Will the adaptation change the core elements of PT? This adaptation does not change the content or delivery of PT, thus preserving the core elements. (6) When will the adaptation be made in the implementation process? Engaging leadership and administration in the implementation support of PT can occur before the groups begin (pre-implementation and planning) to set expectations, and during implementation of PT when children are unable to pay for school fees and attend PT. (7) Who will be impacted by the adaptation? Children engaged in PT will be directly impacted as they will be permitted to remain in school and PT sessions. (8) Who is involved in executing the adaptation? Will all perspectives be considered? Teachers will be involved in executing the adaptation regardless of whether the group is conducted by CHVs or teachers, as teachers have an existing relationship with school leadership. In addition, school leaders and administrators will play an important role in allowing fee waivers and postponement.

Based on the above eight questions adapted from the FRAME, we proposed two additional questions. (9) Is this adaptation feasible? Often, schools have connections to other resources that can ultimately support the children receiving PT. However, without communication and collaboration, the school administration may not know who needs the resources or support. This strategy relies on existing relationships between counselors and school administration and does not require guardians to spend their own money to support children. (10) Is this adaptation impactful? Through this strategy, guardians and children may be given more time and/or financial resources to address their SRFs that impact their attendance and engagement in school, and thus PT. However, the ultimate impact relies on whether leadership and administration are willing to make exceptions to the rules. In addition, while the strategy may mitigate the effect of the SRFs in the moment, it may not be a sustainable strategy that teaches children and guardians how to address other SRFs when they may arise in the future. Thus, additional strategies may need to be considered here to more holistically support children and guardians.

#### Economic empowerment.

Economic empowerment or gaining education in how to manage finances and how to generate income, was highlighted by counselors and guardians as a strategy that can have lasting impact on addressing SRFs. (1) Why do you need to make an adaptation? This adaptation can target the majority of SRFs that guardians and counselors reported as it allows children and guardians to engage in profitable activities and financially support themselves. (2) What type of adaptation is this? This adaptation involves adding elements to the PT protocol as counselors teach children and guardians how to engage in income generating activities. (3) Has this adaptation been made proactively or re-actively before? What are lessons learned? Although not done systematically, counselors reported that some counselors helped guardians work together, pool resources, and develop an income generating activity together. No other counselor reported how economic empowerment was taught in PT, thus this adaptation needs additional refinement. (4) Is the adaptation changing the content, how the treatment is delivered, the training in the treatment, or the treatment implementation? This adaptation may require additional training in how to teach economic empowerment to children and/or guardians. In addition, depending on what types of strategies are taught to increase economic empowerment, the delivery of treatment may be changed (e.g., meeting in the school garden instead of the classroom). (5) Will the adaptation change the core elements of PT? This adaptation does not remove any core elements of PT. (6) When will the adaptation be made in the implementation process? The planning for this adaptation can occur during the implementation phase and in the sustainment phase as economic empowerment can be incorporated during PT and after PT is complete. (7) Who will be impacted by the adaptation? The children and guardians enrolled in PT and their families will likely have a positive impact. Counselors may experience increased burden to support economic empowerment. (8) Who is involved in executing the adaptation? Will all perspectives be considered? Counselors will likely lead this adaptation, and guardians and other individuals with resources in the community may be included if willing to support the adaptation. The economic empowerment strategies can be adapted based on the needs and resources of the community and should consider all perspectives during planning of the adaptation. (9) Is this adaptation feasible? As this adaptation can take place during PT and does not necessitate money, the adaptation is likely feasible. In communities with fewer existing resources and lower engagement from those who have resources, the feasibility may be lower. (10) Is this adaptation impactful? This adaptation has potential to generate revenue for children and families and support nutrition, academics, and engagement in PT. Further, counselors reported seeing positive impact following the implementation of similar strategies alongside PT.

Through economic empowerment, guardians and children can learn how to harness existing resources in their community and work with their peers to generate income and support their family. This can lead to increased money, food, and resources that ultimately will help children and guardians directly address the SRFs that are impacting their life and engagement in PT. Example activities that counselors and guardians described were growing a tree nursery, learning to use innovative farming techniques (e.g., using plastic bags to grow kale), and chicken rearing. Although the activity will likely differ based on the context, the methods used to identify the need and plan the activity will likely follow a similar format that can be shared within PT training and supervision and generalized beyond PT and the western Kenyan context. Counselors would not be expected to contribute money, and instead teach children and guardians to utilize methods in identifying and planning economic empowerment strategies alongside PT.

### Implications and future research

Results from this project can inform future development of strategies to address SRFs through partnered brainstorming workshops and trainings with counselors and PT supervisors. We presented this information to our partners and systematically developed strategies to address SRFs in therapy in a subsequent study. We focused on targeting the most feasible and most impactful strategies described in this manuscript. Further, despite the project originating from a community need, approaches to address SRFs can be expanded beyond western Kenya. The types of SRFs may be different, though the impact on mental health care is likely to be similar. We encourage other researchers and clinics to work alongside partners to understand the specific needs of a community and whether there are opportunities to make adaptations to mental health care with the aim of better serving clients and their families.

### Limitations

It is important to consider the limitations of this study. Although every effort was made to reduce possible power differentials within the research and community partnership context, the principal investigator is a white woman from a large University in the United States, and the funding is US-based. Despite working alongside a research and coding team of majority Kiswahili speakers, the first author/principal investigator of this study does not have lived experience in western Kenya or speak Kiswahili. Thus, there may be nuance in the data that was missed or overlooked, despite careful consideration. In addition, it is possible that certain information was missed during the data coding stage as we utilized the detailed notes of the interviews, written by interview note-takers rather than the audio recordings. Another limitation inherent in qualitative research is social desirability, such the participants may have provided responses that were perceived as socially appropriate to the interviews. To mitigate the presence of social desirability, interviewers were members of the community and spoke the language of the participants. The strategies developed in this research also likely reflect the strengths and needs of Bungoma County, the fifth largest county in Kenya and may not reflect those of other communities worldwide. However, we found heterogeneity between schools within Bungoma county and therefore focused on ways to support lay providers in adapting treatments for their own community and needs, and this process may be helpful in other regions. Furthermore, we did not ask participants or analyze the connections between types of SRFs, the strategies used, and impacts of addressing those SRFs. In future research, it would be important to identify the connections so that counselors can recognize which strategy would be most feasible and impactful depending on the specific type of SRFs they observed.

## Conclusion

In response to calls to mitigate effects of SRF in mental health care, the current study investigated the impact of SRFs on mental health and therapy engagement and identified potential strategies to address SRFs within group therapy in western Kenya. Given the widely reported negative impact of SRFs on mental health care and engagement, participants in this study were already addressing the SRFs within therapy implementation and reported positive impact on mental health and treatment engagement. However, most strategies already in use relied on counselors’ personal money and resources. Thus, we highlight the utility in guiding the development and implementation planning of strategies through an overarching framework, the adapted-FRAME, to ensure the strategies are feasible, impactful, and ultimately remove the undue financial burden on mental health counselors.

## Supporting information

S1 Text Appendix A. Qualitative interview guides. (DOCX)

S2 Text Appendix B. Example STEP sheet that incorporate stigma psychoeducation and coping with stigma. (DOCX)

S1 COREQ Checklist COREQ (COnsolidated criteria for REporting Qualitative research) checklist. (PDF)

## References

[1] Cohen JA, Mannarino AP, Deblinger E. Treating trauma and traumatic grief in children and adolescents. Guilford Publications; 2016.

[2] Ritchie H, Roser M. Mental health. Our world in data. [cited 2018 May];19:2020.

[3] World Health Organization. Department of Mental Health, Substance Abuse, World Health Organization, World Health Organization. Department of Mental Health, Substance Abuse. Mental Health, World Health Organization. Mental Health Evidence, et al. Mental health atlas 2005. World Health Organization; 2005.

[4] Chisholm D, Burman-Roy S, Fekadu A, Kathree T, Kizza D, Luitel NP, et al. Estimating the cost of implementing district mental healthcare plans in five low- and middle-income countries: the PRIME study. Br J Psychiatry. 2016;208 Suppl 56(Suppl 56):s71-8. doi: 10.1192/bjp.bp.114.153866 26447170 PMC4698559

[5] Moitra M, Owens S, Hailemariam M, Wilson KS, Mensa-Kwao A, Gonese G, et al. Global mental health: where we are and where we are going. Curr Psychiatry Rep. 2023;25(7):301–11. doi: 10.1007/s11920-023-01426-8 37256471 PMC10230139

[6] Osazuwa S, Moodley R. Will there be a willingness to actually engage with it?: Exploring attitudes toward culturally integrative psychotherapy among Canada’s African community. J Psychother Integr. 2023;33(1):68–85.

[7] Lovero KL, Dos Santos PF, Come AX, Wainberg ML, Oquendo MA. Suicide in global mental health. Curr Psychiatry Rep. 2023;25(6):255–62. doi: 10.1007/s11920-023-01423-x 37178317 PMC10182355

[8] Rose-Clarke K, Bitta M, Evans-Lacko S, Jokinen T, Jordans M, Nyongesa MK, et al. Centring youth mental health discourse on low-income and middle-income countries. Lancet Psychiatry. 2024;11(9):671–2. doi: 10.1016/S2215-0366(24)00211-6 39147454

[9] World Health Organization. Closing the gap in a generation: health equity through action on the social determinants of health: Commission on Social Determinants of Health final report. World Health Organization; 2008. 10.1016/S0140-6736(08)61690-6 18994664

[10] Bender K, Brown SM, Thompson SJ, Ferguson KM, Langenderfer L. Multiple victimizations before and after leaving home associated with PTSD, depression, and substance use disorder among homeless youth. Child Maltreat. 2015;20(2):115–24. doi: 10.1177/1077559514562859 25510502

[11] Martin MS, Maddocks E, Chen Y, Gilman SE, Colman I. Food insecurity and mental illness: disproportionate impacts in the context of perceived stress and social isolation. Public Health. 2016;132:86–91. doi: 10.1016/j.puhe.2015.11.014 26795678

[12] Fleury M-J, Ngui AN, Bamvita J-M, Grenier G, Caron J. Predictors of healthcare service utilization for mental health reasons. Int J Environ Res Public Health. 2014;11(10):10559–86. doi: 10.3390/ijerph111010559 25321874 PMC4210995

[13] Abrams EM, Greenhawt M, Shaker M, Pinto AD, Sinha I, Singer A. The COVID-19 pandemic: adverse effects on the social determinants of health in children and families. Ann Allergy Asthma Immunol. 2022;128(1):19–25. doi: 10.1016/j.anai.2021.10.022 34699969 PMC8539831

[14] UNICEF. The state of the world’s children: 2009: maternal and newborn health [Internet]. New York; 2008. Available from: https://www.unicef.org/sowc09/

[15] UNICEF. The state of the world’s children 2015: reimagine the future. [Internet]. New York; 2015. Available from: http://sowc2015.unicef.org/

[16] Lee VC, Muriithi P, Gilbert-Nandra U, Kim AA, Schmitz ME, Odek J, et al. Orphans and vulnerable children in Kenya: results from a nationally representative population-based survey. J Acquir Immune Defic Syndr. 2014;66 Suppl 1(Suppl 1):S89-97. doi: 10.1097/QAI.0000000000000117 24732824 PMC4794990

[17] UNICEF. UNICEF Data: monitoring the situation of children and women. Global and regional trends [Internet]; 2024. Available from: https://data.unicef.org/topic/hivaids/global-regional-trends/

[18] Atwine B, Cantor-Graae E, Bajunirwe F. Psychological distress among AIDS orphans in rural Uganda. Soc Sci Med. 2005;61(3):555–64. doi: 10.1016/j.socscimed.2004.12.018 15899315

[19] Okawa S, Yasuoka J, Ishikawa N, Poudel KC, Ragi A, Jimba M. Perceived social support and the psychological well-being of AIDS orphans in urban Kenya. AIDS Care. 2011;23(9):1177–85. doi: 10.1080/09540121.2011.554530 21500024

[20] Puffer ES, Drabkin AS, Stashko AL, Broverman SA, Ogwang-Odhiambo RA, Sikkema KJ. Orphan status, HIV risk behavior, and mental health among adolescents in rural Kenya. J Pediatr Psychol. 2012;37(8):868–78. doi: 10.1093/jpepsy/jss077 22728899 PMC3437686

[21] Cherewick M, Dahl RE, Bertomen S, Hipp E, Shreedar P, Njau PF, et al. Risk and protective factors for mental health and wellbeing among adolescent orphans. Health Psychol Behav Med. 2023;11(1):2219299. doi: 10.1080/21642850.2023.2219299 37274749 PMC10234133

[22] Mishra V, Arnold F, Otieno F, Cross A, Hong R. Education and nutritional status of orphans and children of HIV-infected parents in Kenya. AIDS Educ Prev. 2007;19(5):383–95. doi: 10.1521/aeap.2007.19.5.383 17967109

[23] Mwaniki EW, Makokha AN, Muttunga JN. Nutrition status and associated morbidity risk factors among orphanage and non-orphanage children in selected public primary schools within Dagoretti, Nairobi, Kenya. East Afr Med J. 2014;91(9):289–97. 26866080

[24] Bryant M, Beard J. Orphans and vulnerable children affected by human immunodeficiency virus in Sub-Saharan Africa. Pediatr Clin North Am. 2016;63(1):131–47. doi: 10.1016/j.pcl.2015.08.007 26613693

[25] Dorsey S, Lucid L, Martin P, King KM, O’Donnell K, Murray LK, et al. Effectiveness of task-shifted trauma-focused cognitive behavioral therapy for children who experienced parental death and posttraumatic stress in Kenya and Tanzania: a randomized clinical trial. JAMA Psychiatry. 2020;77(5):464–73. doi: 10.1001/jamapsychiatry.2019.4475 31968059 PMC6990668

[26] Jeste DV, Pender VB. Social determinants of mental health: recommendations for research, training, practice, and policy. JAMA Psychiatry. 2022;79(4):283–4. doi: 10.1001/jamapsychiatry.2021.4385 35195678

[27] Centers for Disease Control and Prevention. Social determinants of health: know what affects health [Internet]; 2021. Available from: https://www.cdc.gov/socialdeterminants/index.htm

[28] Becker CB, Middlemass K, Taylor B, Johnson C, Gomez F. Food insecurity and eating disorder pathology. Int J Eat Disord. 2017;50(9):1031–40. doi: 10.1002/eat.22735 28626944

[29] Paananen R, Ristikari T, Merikukka M, Gissler M. Social determinants of mental health: a Finnish nationwide follow-up study on mental disorders. J Epidemiol Community Health. 2013;67(12):1025–31. doi: 10.1136/jech-2013-202768 23908462

[30] Cluver LD, Gardner F, Operario D. Effects of stigma on the mental health of adolescents orphaned by AIDS. J Adolesc Health. 2008;42(4):410–7. doi: 10.1016/j.jadohealth.2007.09.022 18346667

[31] Hadley C, Patil CL. Food insecurity in rural Tanzania is associated with maternal anxiety and depression. Am J Hum Biol. 2006;18(3):359–68. doi: 10.1002/ajhb.20505 16634017

[32] Anderson SA, Jackson LC. Training and supervision needs of practitioners working with African American women. Women Ther. 2019;42(3–4):469–91. doi: 10.1080/02703149.2019.1622906

[33] Patallo BJ. The multicultural guidelines in practice: cultural humility in clinical training and supervision. Train Educ Prof Psychol. 2019;13(3):227.

[34] Utsey SO, Gernat CA, Hammar L. Examining white counselor trainees’ reactions to racial issues in counseling and supervision dyads. Couns Psychol. 2005;33(4):449–78.

[35] Gantt AC, Johnson KF, Preston JW, Suggs BG, Cannedy M. School counseling interns’ lived experiences addressing social determinants of health. Teach Superv Couns. 2021;3(3):7.

[36] Johnson KF, Cunningham PD, Tirado C, Moreno O, Gillespie NN, Duyile B, et al. Social determinants of mental health considerations for counseling children and adolescents. J Child Adolesc Couns. 2023;9(1):21–33. doi: 10.1080/23727810.2023.2169223

[37] Neal KS, Coleman ML, Hicks Becton LY, Springfield J. Assessing the social determinants of mental health in counseling practice. J Couns Dev. 2023;101(4):381–91.

[38] Pester DA, Jones LK, Talib Z. Social determinants of mental health: informing counseling practice and professional identity. J Couns Dev. 2023;101(4):392–401.

[39] Gantt‐Howrey A, Brookover DL, Robins LB. Addressing social determinants of mental health through cognitive behavioral therapy. J Couns Dev. 2025;103(1):86–96.

[40] Mason S, Ragan M, Gilbert SH, Lenz AS. Social determinants of mental health: implications for measurement, research, and evaluation. J Couns Dev. 2023;101(4).

[41] Gantt‐Howrey A, Lin M, Shaikh A, Johnson KF, Preston JW, Wilson L. Assessing social determinants of mental health: client experiences and counselor practices. J Couns Dev. 2024.

[42] Robins LB, Rodgers D, Barburoglu Y, Griffith J, Arnold CL. Unlocking adolescent mental health: exploring social determinants and depression with the social ecological model. J Hum Serv. 2024;43(1).

[43] Smith L, Il Shin J, McDermott D, Jacob L, Barnett Y, López-Sánchez GF, et al. Association between food insecurity and depression among older adults from low- and middle-income countries. Depress Anxiety. 2021;38(4):439–46. doi: 10.1002/da.23147 33687122

[44] Woan J, Lin J, Auerswald C. The health status of street children and youth in low- and middle-income countries: a systematic review of the literature. J Adolesc Health. 2013;53(3):314-321.e12. doi: 10.1016/j.jadohealth.2013.03.013 23706729

[45] Yeh M, Diaz D, Zerr A, Macias A, McCabe K. Youth-therapist and parent-therapist match and mismatch on internalizing and externalizing treatment goals as predictors of treatment engagement. Adolescents. 2023;3(4):678–92. doi: 10.3390/adolescents3040048 38389932 PMC10883466

[46] Dorsey S, Akiba C, Triplett NS, Lucid L, Carroll H, Benjamin K, et al. Acceptability of Trauma-focused CBT in Western Kenya: a mixed methods study [Manuscript submitted for publication]. 10.1177/26334895221109963 PMC9924250 37091080

[47] Woods-Jaeger B, Cho B, Briggs EC. Training psychologists to address social determinants of mental health. Train Educ Prof Psychol. 2020.

[48] de Haan AM, Boon AE, Vermeiren RRJM, Hoeve M, de Jong JTVM. Ethnic background, socioeconomic status, and problem severity as dropout risk factors in psychotherapy with youth. Child Youth Care Forum. 2014;44(1):1–16. doi: 10.1007/s10566-014-9266-x

[49] de Haan AM, Boon AE, de Jong JTVM, Vermeiren RRJM. A review of mental health treatment dropout by ethnic minority youth. Transcult Psychiatry. 2018;55(1):3–30. doi: 10.1177/1363461517731702 29035137

[50] Armbruster P, Fallon T. Clinical, sociodemographic, and systems risk factors for attrition in a children’s mental health clinic. Am J Orthopsychiatry. 1994;64(4):577–85. doi: 10.1037/h0079571 7847573

[51] Meza RD, Kiche S, Soi C, Khairuzzaman AN, Nales CJR, Whetten K, et al. Barriers and facilitators of child and guardian attendance in task-shifted mental health services in schools in western Kenya. Glob Ment Health (Camb). 2020;7:e16. doi: 10.1017/gmh.2020.9 32742674 PMC7379319

[52] Lund C. Global mental health and its social determinants: how should we intervene? Behav Res Ther. 2023;169:104402. doi: 10.1016/j.brat.2023.104402 37677893 PMC10896750

[53] Lund C, Jordans MJD, Garman E, Araya R, Avendano M, Bauer A, et al. Strengthening self-regulation and reducing poverty to prevent adolescent depression and anxiety: rationale, approach and methods of the ALIVE interdisciplinary research collaboration in Colombia, Nepal and South Africa. Epidemiol Psychiatr Sci. 2023;32:e69. doi: 10.1017/S2045796023000811 38088153 PMC10803189

[54] Proctor E, Silmere H, Raghavan R, Hovmand P, Aarons G, Bunger A, et al. Outcomes for implementation research: conceptual distinctions, measurement challenges, and research agenda. Adm Policy Ment Health. 2011;38(2):65–76. doi: 10.1007/s10488-010-0319-7 20957426 PMC3068522

[55] Cardemil EV. Cultural adaptations to empirically supported treatments: a research agenda. Sci Rev Ment Health Pract. 2010;7(2):8–21.

[56] Castro FG, Barrera M Jr, Holleran Steiker LK. Issues and challenges in the design of culturally adapted evidence-based interventions. Annu Rev Clin Psychol. 2010;6(1):213–39. 20192800 10.1146/annurev-clinpsy-033109-132032 PMC4262835

[57] La Roche MJ, Christopher MS. Changing paradigms from empirically supported treatment to evidence-based practice: a cultural perspective. Prof Psychol Res Pract. 2009;40(4):396.

[58] Lau AS. Making the case for selective and directed cultural adaptations of evidence-based treatments: Examples from parent training. Clin Psychol Sci Pract. 2006;13(4):295.

[59] Wiltsey Stirman S, Baumann AA, Miller CJ. The FRAME: an expanded framework for reporting adaptations and modifications to evidence-based interventions. Implement Sci. 2019;14(1):58. doi: 10.1186/s13012-019-0898-y 31171014 PMC6554895

[60] Day S, Laver K, Jeon Y-H, Radford K, Low L-F. Frameworks for cultural adaptation of psychosocial interventions: a systematic review with narrative synthesis. Dementia (London). 2023;22(8):1921–49. doi: 10.1177/14713012231192360 37515347 PMC10644683

[61] Stirman SW. Implementing evidence-based mental-health treatments: attending to training, fidelity, adaptation, and context. Curr Dir Psychol Sci. 2022;31(5):436–42.

[62] Marques L, Valentine SE, Kaysen D, Mackintosh M-A, Dixon De Silva LE, Ahles EM, et al. Provider fidelity and modifications to cognitive processing therapy in a diverse community health clinic: associations with clinical change. J Consult Clin Psychol. 2019;87(4):357–69. doi: 10.1037/ccp0000384 30883163 PMC6430611

[63] Stirman SW, Miller CJ, Toder K, Calloway A. Development of a framework and coding system for modifications and adaptations of evidence-based interventions. Implement Sci. 2013;8:65. doi: 10.1186/1748-5908-8-65 23758995 PMC3686699

[64] Dorsey S, Gray CL, Wasonga AI, Amanya C, Weiner BJ, Belden CM, et al. Advancing successful implementation of task-shifted mental health care in low-resource settings (BASIC): protocol for a stepped wedge cluster randomized trial. BMC Psychiatry. 2020;20(1):10. doi: 10.1186/s12888-019-2364-4 31914959 PMC6947833

[65] Cohen JA, Mannarino AP, Deblinger E. Trauma-focused CBT for children and adolescents: treatment applications. Guilford Press; 2012.

[66] O’Donnell K, Dorsey S, Gong W, Ostermann J, Whetten R, Cohen JA, et al. Treating maladaptive grief and posttraumatic stress symptoms in orphaned children in Tanzania: group-based trauma-focused cognitive-behavioral therapy. J Trauma Stress. 2014;27(6):664–71. doi: 10.1002/jts.21970 25418514 PMC4591025

[67] Guest G, Bunce A, Johnson L. How many interviews are enough? An experiment with data saturation and variability. Field Methods. 2006;18(1):59–82.

[68] Hennink M, Kaiser BN. Sample sizes for saturation in qualitative research: a systematic review of empirical tests. Soc Sci Med. 2022;292:114523. doi: 10.1016/j.socscimed.2021.114523 34785096

[69] Dorsey S, Akiba CF, Triplett NS, Lucid L, Carroll HA, Benjamin KS, et al. Consumer perspectives on acceptability of trauma-focused cognitive behavioral therapy in Tanzania and Kenya: a mixed methods study. Implement Res Pract. 2022;3:26334895221109963. doi: 10.1177/26334895221109963 37091080 PMC9924250

[70] Woods-Jaeger BA, Kava CM, Akiba CF, Lucid L, Dorsey S. The art and skill of delivering culturally responsive trauma-focused cognitive behavioral therapy in Tanzania and Kenya. Psychol Trauma. 2017;9(2):230–8. doi: 10.1037/tra0000170 27414470 PMC5237406

[71] Skutnabb-Kangas T. Linguistic genocide in education--or worldwide diversity and human rights? Routledge; 2000.

[72] Braun V, Clarke V. Using thematic analysis in psychology. Qual Res Psychol. 2006;3(2):77–101.

[73] Vindrola-Padros C, Chisnall G, Cooper S, Dowrick A, Djellouli N, Symmons SM, et al. Carrying out rapid qualitative research during a pandemic: emerging lessons from COVID-19. Qual Health Res. 2020;30(14):2192–204. doi: 10.1177/1049732320951526 32865149 PMC7649912

[74] Hamilton A. Qualitative methods in rapid turn-around health services research. Health Serv Res Dev Cyberseminar. 2013;2023:03.

[75] Hamilton AB, Finley EP. Qualitative methods in implementation research: an introduction. Psychiatry Res. 2019;280:112516. doi: 10.1016/j.psychres.2019.112516 31437661 PMC7023962

[76] Eaton K, Stritzke WG, Ohan JL. Using scribes in qualitative research as an alternative to transcription. Qual Rep. 2019;24(3):586–605.

[77] Halcomb EJ, Davidson PM. Is verbatim transcription of interview data always necessary? Appl Nurs Res. 2006;19(1):38–42. doi: 10.1016/j.apnr.2005.06.001 16455440

[78] Tong A, Sainsbury P, Craig J. Consolidated criteria for reporting qualitative research (COREQ): a 32-item checklist for interviews and focus groups. Int J Qual Health Care. 2007;19(6):349–57. doi: 10.1093/intqhc/mzm042 17872937

[79] Duncan S, Pond R. Effective burnout prevention strategies for counsellors and other therapists: a systematic review and meta-synthesis of qualitative studies. Couns Psychol Q. 2024;38(3):526–55. doi: 10.1080/09515070.2024.2394767

[80] Skovholt T, Trotter-Mathison M. The resilient practitioner: Burnout prevention and selfcare strategies for counselors, therapists, teachers, and health professionals. 2nd ed. London, UK: Routledge; 2016.

[81] Castiglione SA. Implementation leadership: a concept analysis. J Nurs Manag. 2020;28(1):94–101. doi: 10.1111/jonm.12899 31705769

[82] Williams NJ, Wolk CB, Becker-Haimes EM, Beidas RS. Testing a theory of strategic implementation leadership, implementation climate, and clinicians’ use of evidence-based practice: a 5-year panel analysis. Implement Sci. 2020;15(1):10. doi: 10.1186/s13012-020-0970-7 32033575 PMC7006179

