# Supplementary material for: Impact of social risk factors on TF-CBT engagement and strategies to mitigate the impact: A qualitative analysis
Source: PLOS Ment Health. 2026 Apr 1;3(4):e0000499. doi: 10.1371/journal.pmen.0000499 (PMC13042626; doi:10.1371/journal.pmen.0000499)
Supplement: S1 Text — (DOCX) [file pmen.0000499.s001.docx]

# **Appendix A. Qualitative Interview Guides**

**Child Interview Guide**

Placement: After social mobility questions

Introduction for children: You may remember that the goal of Pamoja Tunaweza is to support you in feeling better. There are other programs that support children who go through some of the following hardships: ripped uniforms, lack of shoes, lack of food, being mistreated for losing one or two parents, lack of money for school. For children who will go through Pamoja Tunaweza in the future, we want to know how Pamoja Tunaweza could support other hardships like some other programs do.

1. Thinking back to when you received the Pamoja Tunaweza program, did Pamoja Tunaweza Counselors help support children who experienced these or other hardships?
   1. (yes/no)
      1. If Yes: How?
      2. [If no, provide the following examples of those hardships mentioned above - torn uniforms, lack of shoes, lack of food, being mistreated for losing one or two parents, lack of money for school – and ask question one more time.]
2. For a child that will receive Pamoja Tunaweza in the future, what do you wish Pamoja Tunaweza counselors could say or do to benefit children experiencing these hardships?
   1. [if they have not mentioned financial related support, probe for financial related support] For a child that will receive Pamoja Tunaweza in the future, what do you wish CHV/Teacher counselors could say or do to benefit children experiencing financial related hardships like lack of school fees, uniform, food etc.] What do you wish Pamoja Tunaweza counselors could say or do for that child?”
   2. [if they have not mentioned support related to stigma, probe for support related to stigma] For a child that will receive Pamoja Tunaweza in the future, what do you wish CHV/Teacher counselors could say or do to benefit children who are made fun of or treated differently because of losing a parent?

**Guardian Interview Guides**

Questions to be added to the guardian interview:

Introduction for guardians: You may remember that the goal of Pamoja Tunaweza is to support children in feeling better. There are other programs that support children who go through some of the following hardships: ripped uniforms, lack of shoes, lack of food, being mistreated for losing one or two parents, lack of money for school. For children and guardians who will go through Pamoja Tunaweza in the future, we want to know how Pamoja Tunaweza could support other non-mental health needs like some other programs do.

1. Did your child or other children experience any of these hardships or others we didn’t mention?
   1. If yes: What did they experience? [if no, repeat the hardships mentioned above and listed below]
      1. Torn uniforms
      2. Lack of shoes
      3. Lack of food
      4. Being mistreated for losing one or two parents
      5. Lack of money for school
      6. Other
2. Did these hardships get in the way of attending or learning from Pamoja Tunaweza lessons?
   1. If yes: How?
   2. Probe: In question, you said your or another child experienced (Did this hardship get the way?
3. Apart from helping your child feel better, did Pamoja Tunaweza counselors help support or address hardships that we talked about?
   1. [If yes]:
      1. What did they do?
      2. Did these supports continue during and after Pamoja Tunaweza? What kept them going or stopped the supports?
      3. Did this support help or encourage guardians and children to attend more lessons and participate more in Pamoja Tunaweza?
   2. Probe: In question, you said your or another child experienced (Did this hardship ask this question).
   3. – make it clear on the sheet --
4. If counselors had another opportunity to provide Pamoja Tunaweza again to children and guardians in the future, how would you want them to support different hardships children and guardians may face?
   1. Remind them about the hardships that they’ve already mentioned
   2. Probe that they can’t, not limiting them with feasibility/doability – just explain every possible
   3. For clarity, include hardships: so the interviewer can use: remind the participants hardships mentioned earlier
   4. How would this help a child or guardians? [keep open] – when they mention something that is not related to Pamoja Tunaweza probe how would help a child
5. More generally, are there things that you wish you or your child would have spent time on or learned during Pamoja Tunaweza lessons?
   1. If No: bring up question on example (praise) and some other

____ **Notes reconciled** ____ **Scanned** ____ **Audio uploaded**

*Status (check when complete):*

**Interviewer:** _____________________________

**Guardian Study ID:** __--__ __ __--__ __--__

**Date of interview** (DD/MM/YYYY): __ __ / __ __ / __ __ __ __

*Fill at time of data collection:*

*Guardian Version*

*English*

**BASIC STUDY**

**SOCIAL RISK FACTORS**

**QUALITATIVE INTERVIEW**

*Notes:*

INSTRUCTIONS FOR PARTICIPANT:

**BASIC Semi-Structured Social Risk Factors Qualitative Interview – Guardian Version**

*Thank you for being willing to do an interview with us* *You may remember that the goal of Pamoja Tunaweza is to support children in feeling better. There are other programs that support children who go through some of the following hardships: torn uniforms, lack of shoes, lack of food, being mistreated for losing one or two parents, lack of money for school. For children and guardians who will go through Pamoja Tunaweza in the future, we want to know how Pamoja Tunaweza could support other non-mental health needs like some other programs do.*

***Interviewer note:*** *Throughout the interview, please remind the participant not to mention any specific names but to specify roles (e.g., guardian counselor, child counselor).*


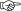


1. **Did your child or other children experience any of these hardships or others we didn’t mention?** (Circle answer)
   1. **Yes**
   2. **No**

*[Probe if no]:* **Did your or other children experience torn uniforms, lack of shoes, lack of food, being mistreated for losing one or two parents, lack of money for school?**

*[Probe if yes]:* **What did they experience?** (Circle answers)

- - 1. Lack of food/hunger
    2. Lack of school fees
    3. Lack of exam fees
    4. Treated differently because of losing one or two parents
    5. Ripped uniform
    6. Lack of shoes
    7. Other (please describe):

1. **Did these hardships get in the way of attending or learning from Pamoja Tunaweza lessons?** (Circle answer)
   1. **Yes**
   2. **No**

*[Probe if no]:* **In the last question you mentioned the following hardships** (list the hardships mentioned before). **Did those hardships get in the way of attending or learning from Pamoja Tunaweza lessons?**

*[Probe if yes]:* **How?**

***Interviewer note:***

***Consider using these probes if needed***

- *Some general probes: “Tell me more about that”, “Can you give me an example?”, “What else?”, “Anything else you would like to add?”*
- *Use the following probes when needed to get specific details: WHAT, HOW, WHEN, HOW OFTEN, BY WHOM.*


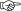


1. **Apart from helping your child feel better, did Pamoja Tunaweza counselors help support or address hardships that we talked about? (**Circle answer)
   1. **Yes**
   2. **No**

*[Probe if no]:* **In the first question you mentioned the following hardships** (list the hardships mentioned before). **Did Pamoja Tunaweza counselors help support or address those hardships?**

*[Probe if yes]:* **What did they do?**

*Follow up question:* **Did these supports continue during and after Pamoja Tunaweza? What kept them going or stopped the supports?**

*Follow up question:* **Did this support help or encourage guardians and children to attend more lessons and participate more in Pamoja Tunaweza?**

1. **If counselors had another opportunity to provide Pamoja Tunaweza again to children and guardians in the future, how would you want them to support different hardships children and guardians may face?**

*[probe if unsure]:* **You may think of any type of support with no limits for these hardships.**

*[probe if unsure]:* **In the first question you mentioned the following hardships** (list the hardships mentioned before). **How would you want CHV/Teacher counselors to support those hardships?**

**Follow up question**: **How would this help a child or guardians?**

***Interviewer note:***

- *Some general probes: “Tell me more about that”, “Can you give me an example?”, “What else?”, “Anything else you would like to add?”*
- *Use the following probes when needed to get specific details: WHAT, HOW, WHEN, HOW OFTEN, BY WHOM.*


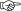


1. **More generally, are there things that you wish you or your child would have spent time on or learned during Pamoja Tunaweza lessons?**

*[Probe if no]* **For example, a topic that you learned about in Pamoja Tunaweza was about praise. What type of topics do you wish you learned about or spent more time on during Pamoja Tunaweza lessons?**

***Interviewer note:***

- *This question is very open and general, let the guardian answer however they would like.*
- *Some general probes: “Tell me more about that”, “Can you give me an example?”, “What else?”, “Anything else you would like to add?”*
- *Use the following probes when needed to get specific details: WHAT, HOW, WHEN, HOW OFTEN, BY WHOM.*


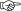


***BEFORE YOU END THE INTERVIEW (if applicable):***

**Interviewer review notes and see if there is anything else that you need to follow-up on.**


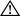


**Additional Space**

*Counselor Version*

*English*

**BASIC STUDY**

**SOCIAL RISK FACTORS**

**QUALITATIVE INTERVIEW**

____ **Notes reconciled** ____ **Scanned** ____ **Audio uploaded**

**Interviewer (circle if this was your role):** _____________________________

**Counselor Study ID:** __--__ __ __--__ __--__

**Date of interview** (DD/MM/YYYY): __ __ / __ __ / __ __ __ __

*Notes:*

*Status (check when complete):*

*Fill at time of data collection:*

**BASIC Semi-Structured Social Risk Factors Qualitative Interview – Counselor Version**

INSTRUCTIONS FOR PARTICIPANT:

*Congratulations on doing such a great job providing Pamoja Tunaweza! We have seen and heard of the great things you have done with your co-counselors. We’ve heard from Pamoja Tunaweza counselors that children who received Pamoja Tunaweza often attend lessons with non-mental health needs like torn uniforms, no shoes, difficulty paying for exam fees, being hungry, and being treated differently because of their orphan status to name a few. As you know, the goal of Pamoja Tunaweza is to help children feel better after the loss of the parents and it is not an expectation or prescribed to address these hardships. However, some counselors wished that the Pamoja Tunaweza program offered support for these hardships. We are hoping to learn how Pamoja Tunaweza can better support children in these hardships and would like your help. Although it’s not a part of the Pamoja Tunaweza, we know that some counselors offered support for these hardships. So, we’d like to know what you or other counselors may have done to support these hardships and what ideas you might have for bringing more of this support into Pamoja Tunaweza in the future.*

***Interviewer note:*** *Throughout the interview, please remind the participant not to mention any specific names but to specify roles (e.g., child, guardian).*


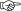


1. **During Pamoja Tunaweza:**
   1. **What sort of hardships did guardian and children talk about in the lessons or did you observe among the children (i.e., did children mention things that disturbed them at home and at school)?**

(Circle answers)

- - - 1. **Lack of food/hunger**
      2. **Lack of school fees**
      3. **Lack of exam fees**
      4. **Treated differently because of losing one or two parents**
      5. **Torn uniform**
      6. **Lack of shoes**
      7. **Other (please describe):**

*[If not mentioned, probe:]* **Did this affect the child or guardian at home? Did this affect the child at school?**

- 1. **Please describe examples of how the children and/or guardian discussed their hardships.**

[*probe if cannot answer*]: **You mentioned that children and guardians experience these hardships** (*insert hardships mentioned above).* **Can you please give me an example of how the child or guardian discussed these hardships with you?**

*[Probe if appropriate]:* **How did other Pamoja Tunaweza children and/or guardians respond when these hardships were discussed?**

***Interviewer note:***

***Consider using these probes if needed***

- *Some general probes: “Tell me more about that”, “Can you give me an example?”, “What else?”, “Anything else you would like to add?”*
- *Use the following probes when needed to get specific details: WHAT, HOW, WHEN, HOW OFTEN, BY WHOM.*


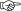


1. **Think of the hardships mentioned above.**
   1. **Did these hardships get in the way of children and guardian’s participation in Pamoja Tunaweza?** *(Circle answer)*
      - 1. **Yes**
        2. **No**

*[If yes, probe:]* **How?**

*[If no, probe:]* **the hardships you mentioned are** *(insert hardships mentioned above).* **Did these hardships get in the way of children and guardian’s participation in Pamoja Tunaweza?**

**Follow up question**: **Je hii ingesaidia kivipi mtoto au walezi?**

***Interviewer note:***

- *Some general probes: “Tell me more about that”, “Can you give me an example?”, “What else?”, “Anything else you would like to add?”*
- *Use the following probes when needed to get specific details: WHAT, HOW, WHEN, HOW OFTEN, BY WHOM.*


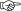


1. **During Pamoja Tunaweza delivery, were there times when you or other counselors referred or supported children and guardians who were experiencing these hardships?** (*Circle answer*)
   - - 1. **Yes**
       2. **No**

*[if no, probe]:* **The hardships that you mentioned are** (*insert hardships mentioned in #1).* **Were there times when you or other counselors referred or supported children and guardians who were experienced** *(Insert hardships mentioned)***?**

*[if yes, probe]:*

**i. Please describe.**

**ii.** *[Probe if not mentioned yet*] **Please describe how this support helped children.**

**iii.** *[Probe if not mentioned yet]***: Please describe how this support helped guardians.**

**iv. Where there any barriers to providing this support or continuing to provide this support?**

*[if yes]* **Please explain.**

*[If no, probe:]* **The supports you mentioned that you provided are** *(insert supports mentioned above in i.).* *Ask iv. again.*

1. **What do you wish was included in the 6-day Pamoja Tunaweza training that you had before starting the lessons that would help in addressing the hardships during Pamoja Tunaweza?**

*[Probe if needed]:*  **Remember that the 6-day training included topics like the step sheets for each lesson, building interest among children and guardians, observation of trainers modeling skills, and counselor skill practice in small groups. Apart from what was covered in the 6-day Pamoja Tunaweza training, what do you wish was added to the training that would help in addressing these hardships during Pamoja Tunaweza?**

*[Probe:]* **Think about other programs that you know about where the focus is on these hardships.**

1. **If you or other Pamoja Tunaweza counselors had more time and resources, what could Pamoja Tunaweza counselors do to help children and guardians with these hardships?**

***BEFORE YOU END THE INTERVIEW (if applicable):***

**Interviewer to check if there is anything else that the interviewer needs to follow-up on.**


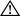


**Additional Space**
