# Supplementary material for: Impact of social risk factors on TF-CBT engagement and strategies to mitigate the impact: A qualitative analysis
Source: PLOS Ment Health. 2026 Apr 1;3(4):e0000499. doi: 10.1371/journal.pmen.0000499 (PMC13042626; doi:10.1371/journal.pmen.0000499)
Supplement: S2 Text — (DOCX) [file pmen.0000499.s002.docx]

# **Appendix B. Example STEP Sheet that incorporate Stigma Psychoeducation and Coping with Stigma**

*Note.* that the highlighted sections are the adaptations.

**Child Group 1: Introduction and Relaxation**

**Child STEPS**

1. **Welcome/Introduction:**
   - Thank them for agreeing to join the group and praise attendance
   - Remind child about confidentiality agreement.
     - Remind them that you won’t share what they tell you with anyone outside of the Pamoja Tunaweza group unless they say it is ok. Remind them that if there is a threat to them or someone else, you will talk with them about your concerns and make a plan to make sure everyone is safe.
   - Introduction Game – choose one, for example:
     - Use a ball or other way to introduce all in group—Group Leader starts.
     - Play a game with questions you and children answer. Questions may be things like “what is your favorite color,” “what is your favorite food?”...etc.
       - Young children can draw on paper. This could be people, food, colors, etc.
2. **What**

“Today I want to tell you more about the program, talk with you about feelings and problems children may have if a parent died, get to know you a little better, and do something fun together. We are also going to learn about a skill called relaxation.”

1. **Why**

“We will talk about these things because I want to explain a little about feelings, worries, and behaviors children whose parent died might be having, so you will understand you are not alone, if you have had any of these. I also want you to understand that our program helps with these types of feelings and worries. We’ll start one thing that can help today.”

1. **Tell children about the Pamoja Tunaweza program:**

- Our program that helps with EXACTLY these worries and feelings that children have.
- Explain how it has helped other children in Bungoma, Kenya and also in Tanzania, Zambia, and other countries.

“This program has helped many children in Tanzania, Kenya, Zambia, and other countries, too. Children are doing better after receiving this kind of program. That is why we are now expanding to help more children and their guardians here in Bungoma, Kenya.”

Give a Taste of the program

using a triangle!

- Describe that it is a program that works on THOUGHTS and BEHAVIOR.
  - Goal: Learn how we can DO things differently and THINK differently to feel better and feel less worried.
  - GIVE A TASTE OF PROGRAM: Can do an example activity in session to show either doing OR thinking differently (e.g., triangle on a child thinking they will fail an upcoming exam – show original and changed triangle) OR have group tell you how they’re feeling, do a fun activity, and then see how they’re feeling afterwards. Point out the difference.
- **CHOICES FOR GROUP LEADER**: Choose between A and B depending on age of group/children’s attention:
  - - 1. Give a summary overview:

“We will learn skills to relax when stressed, learn how what we think is connected to how we feel and act, learn how to talk about events related to the parent’s death and not feel too stressed or sad, learn how to think about difficult events and situations in a more helpful way, and learn how to feel better about your parent’s death and feel better when you are missing your parent. Today we will start with a skill called relaxation.”

**OR**

- - - “Let me tell you a story about a young elephant, whose mama died. This elephant felt sad, worried that no one would love her like her mama, and worried about who would pay her elephant school fees. She often stayed alone and had trouble in school. Then, she learned about Pamoja Tunaweza. In the program, she learned she was not the only one who’d lost a parent, and she was not alone with her feelings and worries. She learned how to do things to feel better, how to examine her thoughts and change them to feel less worried, and found new ways to remember her mama and to connect to people in her life. After Pamoja Tunaweza, she still missed her mama, but she felt a lot better, less sad, and more connected to people in her home and village.”

1. **Explain the program will take 8 weeks, meeting each week as a group for one hour.**

- Use at least one story to explain why.

“When you take an antibiotic, and you want it to work, can you take it only a few times, and take it whenever you want?”

“When children go to school, can they go only some days, whenever they want and still get their degree? Can they get their degree after only a few days of school, going when they wish?”

“This program is like this story; we need you to come regularly, each week, and participate fully, so that you can feel better.”

Tell what group will be like!

- Tell the children that they will be learning and practicing new skills together throughout the program:
  - In the group
  - At home (as homework)
    - Give an example for why practicing at home is important (practice makes perfect)
      - Learning to ride a bicycle, cook
      - Practice is how you become an expert!
- Explain that you will also be teaching the same skills to the guardians.

1. **Education about grief; normalizing their thoughts, feelings, and behaviors**

- Share some of what we know as common feelings, worries, thoughts & behaviors of children who have had a parent die.

Tell a

Story

and

Use

examples

- Can use story of animal from before…ask children what animal felt and worried about?
- Group leaders name SOME specific feelings/problems/worries.

“Many children whose parent has died say they feel lonely, sad, and unhappy; they have worries about their future; some children sometimes think no one loves them like the parent who died did, they feel Unyanyasaji for losing a parent, and so on. Tell me more about some of the worries you think a child who has lost a parent might have. What are some of the feelings you think a child might have who has lost his/her parent? What might that child then do?”

“Tell me about what orphan stigma means to you. What are some feelings you think a child might have related to stigma and losing his/her parent?”

- - Stigma definition: negative and often unfair beliefs that people have about something or someone. This can lead to being treated differently than others.
  - Normalize, validate and praise as children share ideas.
    - Normalize/validate the experience of stigma/discrimination and tell children that it’s not right/not fair.
  - Encourage children to keep sharing.

1. **Give hope that the program will help.**

“The things we will do in this program have helped many children with problems when they have lost a parent, such as feeling more loved, making more friends, and concentrating better at school. Now they are doing better. This program can help you as well.”

**Now we will talk about RELAXATION:**

1. **What**

“Now we will learn things we can DO to feel better when [NAME FEELINGS CHILDREN NAMED]. “We will do some activities to learn how to help our bodies relax.”

1. **Why**

“Helping our bodies relax physically can help us feel better.”

1. **Link to feelings/worries from the first exercise to illustrate that relaxation will help them.**
2. **Find out what the children do now to reduce stress and anxiety.**

“Are there things that you currently do to relax when you are feeling stressed?”

- Reinforce or praise helpful and appropriate things the children already do to relax (talk to a friend, listen to a song, playing football, singing with friends, playing hide and seek, rope skipping).

1. **Personalize the relaxation topic**

“Today we will work together on some other ways to feel better. You can decide on one of these new skills or something that you already do to practice regularly this week.”

1. **Ask how stressed or anxious the children are feeling.**

“Everyone feels tense or stressed sometimes. How tense/stressed are you feeling right now?”

1. **Introduce and teach the new technique or techniques.**

- Choose askari/acrobat, breathing, or count backward to start.

“There are also new activities that you can do to feel better when you are feeling stressed, such as [CHOOSE ONE TO START].”

1. **Demonstrate one technique—show the children.**
2. **Have the children practice the technique for about 2-3 minutes.**
3. **Ask how tense/anxious the children are feeling now after doing the technique.**

- If the children are feeling more peaceful or less tense or worried, make the connection for the children between their behavior (doing relaxation) and their feelings.

1. **Teach a second new technique.** Do steps 14-17 for this technique.
2. **Have each child choose a skill for the week—either a NEW one or one that already works for the child.**
   - **Make a list of the thing each child chooses to practice.** Share this with the guardian group leader at the end of the group.
3. **Make a plan with the children for using the chosen relaxation skill at times when the children have stress, worries, or sadness related to remembering/memories of the parent’s death.**

“What are times of the day or times during the week that you are reminded of your parent(s)’s death or feel worried, stressed, or sad about your parent(s)’ death?”

- Have each child share one by one.

1. **Brief about Next Group: Cognitive Triangle**

“Next week we will learn about how to THINK in a different way to feel better. We will talk about the special triangle that shows you how thoughts, feelings and behavior are connected.”

1. **Homework: Table of practicing relaxation and their feelings**

- Show the children how to set up a table of their feelings after an event, doing a relaxation exercise, and feelings after the exercise. Give examples; demonstrate by writing on the board and make sure the children understand the assignment:

| **Event/situation** | **Time** | **Feelings** | **Relaxation** | **Feelings after relaxation** |
| --- | --- | --- | --- | --- |
| Late to school | Morning | Sad, fear | Breathing in and out | Less worried. Less sad. |
| Thinking about my mother’s/ father’s death.(we were eating together) | Night | Sad | Askari | Less sad. |
| Student in school making fun of me for my mother’s/  father’s death | School day | Sad, fear | Breathing in and out | Less sad. Less scared. |

- Can encourage children to teach someone else at home—as everyone needs to learn relaxation.
- Tell children that you will ask them to report back the next week on how their practice went.
- **Praise them again for coming to the group and for their participation during the activity---give them thanks.**

Fun Time! Do something fun!

- Ask the children about a fun game to play, or suggest some fun activities (singing a song, playing a game or football etc.). Play for about 2-3 minutes.
- **Children can choose or give ideas:** can be children’s choice.

“We will end each session with something fun we can do together, as a reward for working hard here and practicing new skills, [NAME FUN THINGS CHILDREN LIKE]. Let’s choose a fun activity to do together now.”

***Make sure the guardian group leader knows the relaxation techniques each child chose, so they can inform the child’s guardian.

**Guardian Group 1: Introduction and Relaxation**

**Guardian STEPS**

1. **Welcome/Introduction:**

- The introduction is the same information as in children’s group. Praise them for coming and agreeing to be part of the group and the study.
- Remind guardian about confidentiality agreement.
  - Remind them that you won’t share what they tell you with anyone outside of the Pamoja Tunaweza group unless they say it is ok. Remind them that if there is a threat to them or someone else, you will talk with them about your concerns and make a plan to make sure everyone is safe.
- Give a very warm welcome.

“I am so happy to meet you today and thank you very much for being a part of this program. I know you have a lot to do at home/work but for the betterment of your child you agreed to join this program. We sincerely thank you for the time you are dedicating to this program each week. Thank you very much, and we thank your children also.”

- Group leader and all guardians introduce themselves; share something that helps guardians get to know one another (but is not too personal).
  - Start introduction in fun way that can make a group come together.

1. **What**

“Today I want to tell you more about the program, talk with you about feelings and problems children who have had a parent(s) die might have, and get to know you. I also want to talk to you about relaxation skills to help your children begin to feel better.”

1. **Why**

“I want to explain a little about feelings, worries, and behaviors children who have lost a parent(s) might be having, and understand any of these that your children might be having. I also want to make sure you know what we will be doing in this program together.”

1. **Tell guardians about the Pamoja Tunaweza program**

- Program that helps with the worries and feelings that children have.
- Explain how it has helped many other children in Tanzania, Kenya, Zambia, and other countries, so we are offering it to more children and guardians here in Bungoma.
  - Give examples from previous groups: Many kids are doing better after getting the program; but do not over-promise.

“We had many kids enrolled in this program over the last 8 years and they are doing better. The children who are doing the best are those who both the child and the guardian participated each week and practiced skills at home, without missing any sessions. Their behavior is better now than it was before participating in this program. We would also like to encourage and welcome you to participate fully, with the hopes of similar benefit.”

- 8 group sessions, each 60 minutes; 1-2 individual sessions, depending on the child’s need.
- Describe that it is a program that works on THOUGHTS and BEHAVIOR.
  - Goal: Learn how we can DO things differently and THINK differently to feel better and feel less worried.
  - GIVE A TASTE OF PROGRAM: Do an example activity in session to show *either* doing or thinking differently.

Give a

Taste of the

Program

using a

triangle

- - - THOUGHTS:

Triangle on a child thinking “no one loves me since my baba died” and having group give you feelings and behavior; then CHANGE the thought and other points on the triangle.

**OR**

- - - BEHAVIOR:

Have group tell you how they’re feeling NOW, do a fun activity, ask how they’re feeling NOW after activity.

Choose between A and B below, depending on guardians.

1. Brief Summary of Group

“Guardians and children will learn skills to relax when stressed, learn how what we think is connected to how we feel and act, learn how to talk about events related to the parent’s death and not feel too stressed or sad, learn how to think about difficult events and situations in a more helpful way, and learn how to feel better about the parent’s death and to feel better when the children are missing the parent. Guardians will also learn some specific skills for dealing with children’s behavior—to help with obedience. Only guardians will learn these skills. There will be some sessions where children share with guardians their memories of the parent’s death and what they have learned.”

**OR**

1. Sentence by Sentence Description of each Component

Introduction/Psychoeducation

“First, today, we will talk and learn more about common feelings, worries, and experiences of orphans and learn more about this program—what we’ll do together, what you will learn.”

Relaxation:

“Today you and your children will learn new ways to relax your body and feel better when feeling tense, stressed, or worried. We will talk about different feelings and the strength of feelings in different situations.”

Parenting

“We will learn some skills that will help you with your children’s behavior. Some guardians report that children need help with some behaviors. We have some new tools that will help with these problems and also help children continue to feel better and feel cared for.”

Thinking in a Different Way Part I and Part II

“Next week we will learn about thoughts, feelings, and behavior and how they are connected. We will learn how we can think about situations in a different, more positive way to feel better.”

Trauma Narrative/Talking about Hard Memories:

“I will ask your children to tell me the story (ies) of their parent(s)’ death and surrounding events so that the memories cause less distress and fear when they remember them.”

Conjoint Sessions:

“We will share this story with you, with the child’s permission, so that you can support the child more and understand what he or she remembers.”

Facing Reminders/Triggers:

“Many children are afraid of reminders of the parent(s)’s death and may avoid these reminders, like photos, the grave, and/or the parent’s plates. We will help them slowly face those reminders so they do not have to be afraid anymore.”

Grief Components:

“We will learn some specific ways for your children to remember the parent—to talk about what they (and you) miss about them; things the children still HAVE of their parent that they can call to mind and hold onto, individuals in the children’s lives who care for them and they can connect to, and how to prepare in advance for later times in the children’s lives when they will miss the parent and need extra support.”

1. **Explain why full participation is important.**

- Use a story to explain WHY it is important to attend EACH week.
- Emphasize the importance of full guardian participation EACH week in group AND assisting children with practicing at home.
  - For children to get better, we need the guardians’ full involvement.

“As I told you before, we need your full participation, and your child’s full participation, for your kids to get better.”

“For an example, when a doctor prescribes a medicine, what does he say about it? Does the doctor say you have to take the full dose if you want it to work? Or can you take it only a few times, and stop taking it when you want to?” [*Let guardians reply*]

“Right, those who finish the prescribed dosage get better, compared to those who only take part of the dosage and may NOT feel better or only get a little better.”

“When children go to school, can they go only some days, whenever they want and still get their degree? Can they get their degree after only a few days of school, going when they wish?”

“This program is like this story; we need to meet regularly, each week, for this to work. The 8 weeks are the full dose of the program.”

- Tell the guardians that you will be learning and practicing new skills together throughout the program:
  - In the session
  - At home (as homework)

1. **We will start with Education: Share some of what we know as common feelings, worries, thoughts, and behaviors of children who have had a parent(s) die.**

- Group leaders name SOME specific feelings/problems/worries, including the experience of stigma.
- Ask the group to share more ideas in this area—thoughts, feelings, behaviors, and experiences of stigma that they think children who have lost a parent(s) might have/that they see in their children.
  - Encourage sharing.
- Normalize, validate and praise as guardians share ideas.
- POINT OUT that guardians themselves also often have some of these feelings, worries, and behaviors as they too were close to the parent who died. Point out that they might also experience stigma.

“Tell me more about some of the worries or fears you think a child who has lost a parent might have. What are some of the feelings you think a child might have who has lost his/her parent(s)? What do you see in your children?”

“What are some ways you have seen children who have lost a parent be treated differently than other children? What are some feelings you think a child might have when they are treated differently because they lost his/her parent(s)? What do you see in your children?”

- Share the goal of Education: to normalize /validate children’s and guardians’ feelings/reactions to parent’s death and orphan related stigma.
  - We are teaching this to your children because we want the children to understand that their feelings and problems are normal, they are not the only ones (alone) who experience this kind of problem, and this program will help them to get better.

“This program we will do together will help with children be less sad, show better behavior, make more friends, and concentrate better at school.”

**Now we will talk about RELAXATION:**

1. **What**

“Now we will do some activities together to learn how to relax /reduce feelings of stress so that you can help your children with this. Your children are also learning these skills in their class.”

1. **Why**

“Relaxing can also help us adults feel better, and when we feel better we can help our children better. Learning this will also allow you to help your children practice this important skill.”

1. **Find out what the guardian does now to reduce stress and anxiety.**

“Are there things that you currently do to relax when you are feeling stressed?”

- Reinforce or praise helpful and appropriate things the guardians already do to relax, as they name them (e.g., pray, sing, etc.).

1. **Tell the guardians you will teach a few new skills for relaxation – the same ones you taught the children.**

“Today we will work together on other ways to help reduce stress. You can decide on one that you think may be helpful for you to practice this week. I would also like you to help your child practice one of these skills at home that they have chosen.”

1. **Ask how tense/anxious or stressed the guardians are feeling now.**

“Everyone experiences stress sometimes. How stressed are you feeling right now?”

1. **Introduce and teach the new technique(s).**

- Choose breathing, counting backwards, or askari/acrobat to start – whichever one(s) was taught to the children.
- If the technique your colleague used with the children is a game, you may explain that this is a bit childish but you want the guardians to know and understand what their children worked on.

“This activity may seem a little funny/childish for us to do together, but these are the ones that my colleagues taught the children and I want you to know the same ones. This will also allow you to help your child practice.”

1. **Demonstrate one technique.**
2. **Practice the technique with the guardians for about 2-3 minutes. They can practice in pairs or in the big group.**
3. **Ask how tense/anxious the guardians are feeling now after doing the technique.**

- If the guardians are feeling more peaceful or less tense or worried, make the connection for the guardians between their behavior (doing relaxation) and their feelings.
- If needed, teach a 2^nd^ relaxation technique and do steps 12 through 14 for this technique.

1. **Brief about Next Group**

- Next week the focus will be on: Thinking in a Different Way Part 1 (Cognitive Triangle)
- Guardians will also have a parenting topic: Praise

1. **Practice For This Week**

- Remind that children and guardians will practice at home each week.
- CHILDREN will Practice Relaxation.
- GUARDIANS will Practice Relaxation and help their child practice relaxation;
- Have the guardians work with their children to practice the child’s new relaxation skill.
- Suggest that the guardians set aside time to practice a relaxation skill for themselves. You may explain again that a more relaxed/less tense guardian can help the child also.

1. **Conclude: Depending on the guardians you have, choose whether to do some kind of fun activity at the end or some kind of closing celebration (e.g. Pamoja Tunaweza Song; any other activity or song other….)**
